# Periodontal disease-associated micro-organisms in peri-menopausal and post-menopausal women using or not using hormone replacement therapy. A two-year follow-up study

**DOI:** 10.1186/1472-6831-10-10

**Published:** 2010-04-29

**Authors:** Laura Tarkkila, Kirsti Kari, Jussi Furuholm, Aila Tiitinen, Jukka H Meurman

**Affiliations:** 1 Institute of Dentistry, University of Helsinki, Helsinki, Finland; 2 Department of Obstetrics and Gynaecology, University Central Hospital, Helsinki, Finland; 3 Department of Oral and Maxillofacial Surgery, Helsinki University Central Hospital, Helsinki, Finland

## Abstract

**Background:**

Despite conflicting results on the use of hormone replacement therapy (HRT) there is no doubt that many women benefit from it. Women using HRT are known to be more health conscious in general with putative positive implications in the mouth. However, we observed recently in our cohort hardly any difference in oral health status between HRT-users and non-users. There are only a few studies about HRT and oral microbiota. We hypothesized that counts of periodontal micro-organisms are lower in health-conscious HRT-users than non-users.

**Methods:**

Two-year open follow-up study was conducted on originally 200 HRT-users and 200 non-users from age cohorts of 50-58 years. After clinical examination pooled subgingival plaque samples were taken for polymerase chain reaction analyses. The results of finally 135 women meeting the inclusion criteria were analyzed with cross-tabulation and chi-square test. Explanatory factors were studied by step-wise logistic regression analysis.

**Results:**

In HRT group, the numbers of positive samples for *Porphyromonas gingivalis (P. gingivalis*, p < 0.07), *Prevotella intermedia *(*P. intermedia*, p < 0.05)and *Tannerella forsythia *(*T. forsythia*, p < 0.01) decreased in women with ≥ 4-mm-deep pockets. Respectively in HRT users with ≥ 6-mm-deep pockets the numbers of positive samples for *P. gingivalis *(p < 0.05) and *T. forsythia *(p < 0.01) were decreased. No corresponding differences were observed in the non-HRT group. In logistic regression, the existence of deep periodontal pockets explained the majority of cases harboring specific micro-organisms in both groups.

**Conclusion:**

Although use of HRT did not correlate with periodontal health status, HRT led to decreasing numbers of positive samples of the periodontal pathogens *P. gingivalis *and *T. forsythia*. Further studies with longer observation time are needed to observe the clinical relevance of the results.

## Background

Women use hormone replacement therapy (HRT) to cope with estrogen deficiency-induced vasomotor and urogenital symptoms. Women do not only take HRT to avoid climacteric symptoms but also to maintain quality of life and to prevent osteoporosis [1,2]. The use of HRT has received much publicity, however, when the results from large randomized controlled trials were published [3-6]. Recent experimental and clinical studies have indicated that effects of HRT depend on the estrogen and progesterone/progestin formulation, dosage, mode of administration, patient's age, associated diseases, and duration of treatment [7,8]. However, there is no doubt that many women clearly benefit from the use of HRT, which may also have implications in the oral cavity [9].

Chronic infections are involved in the etiopathogenesis of many systemic diseases by releasing pro-inflammatory mediators that may cause endothelial damage and initiate, for example, cholesterol plaque attachment (for review, see [10]). Periodontal disease being highly prevalent in the populations presents a marked inflammatory burden in this regard (for review, see [11]).

There is little knowledge about the prevalence of periodontal microbiota among peri-menopausal and post-menopausal women [12]. However, sex hormones have long been considered to play an influential role on periodontal tissues and periodontal disease progression and therefore the role of sex hormones has been of interest in further investigations [13-16]. We thought it interesting to study if differences occur in harboring certain periodontal bacteria, namely *Porphyromonas gingivalis *(*P. gingivalis*), *Tannerella forsythia *(*T. forsythia*), *Aggregatibacter actinomycetemcomitans *(*A. actinomycetemcomitans*), *Treponema denticola *(*T. denticola*), *Prevotella intermedia *(*P. intermedia*) and *Prevotella nigrescens *(*P. nigrescens*), between pre- and postmenopausal women using or not using HRT. The follow-up time was two years and the present report is a continuation of our study on oral health of the cohort of peri-menopausal and postmenopausal women in the age groups of 50-58 years [17]. In the earlier study we did not observe any difference in the clinical oral health status between the groups. However, taking into account the known associations between periodontal bacteria and systemic diseases [10] and the interactions between HRT use and general health [6] we thought that HRT might affect periodontal infections as reflected in the occurrence of specific bacteria. We thus assumed that the more health-conscious HRT users harbor less frequently periodontal pathogens when compared with non-users at the end of this 2-year study.

## Methods

### Patients and their examination

Details of our patient material are given in our earlier contribution [17]. Patients were chosen from original sample of 3173 women at age cohorts 50, 52, 54, 56, and 58 years, who had participated in our questionnaire study on oral symptoms [18]. Of those five age cohorts 40 women reporting the use of HRT and 40 reporting not to use HRT at the time of the questionnaire were randomly selected and invited to attend our clinical study. Hence, 200 HRT users and 200 non-HRT users received our invitation letter. One recall letter was sent to those who did not respond to the first invitation.

**Figure 1:**
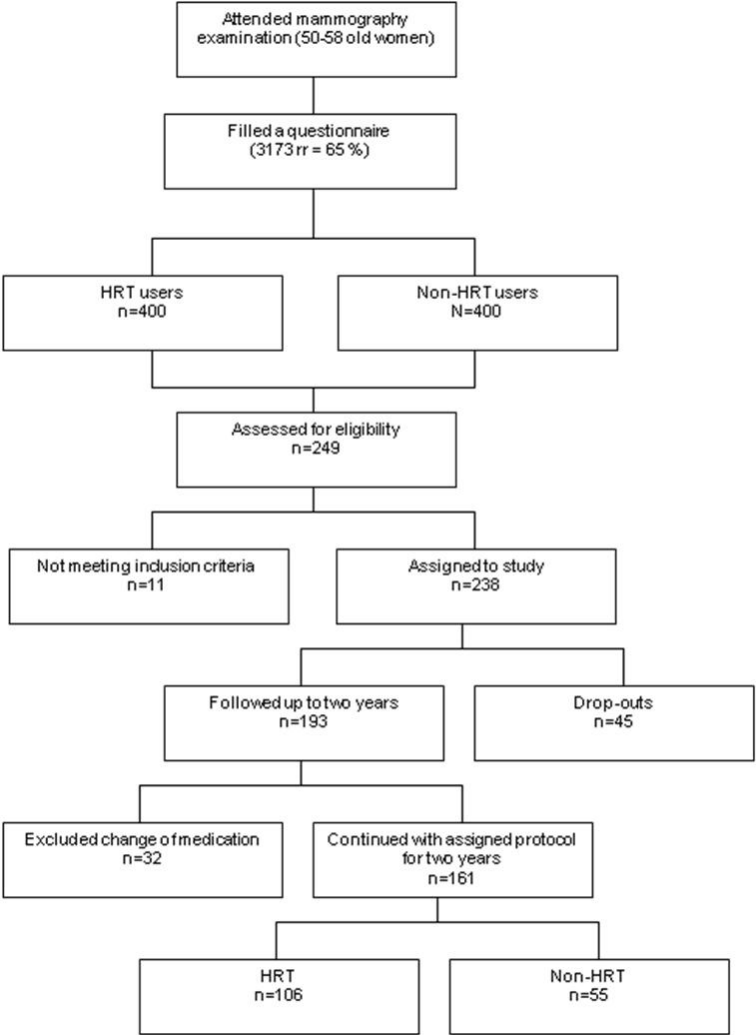
**Flow-chart of the inclusion of subjects**.

For the baseline study altogether 249 women attended, with a response rate of 62%. After two years all those who attended the baseline clinical study were invited to a follow-up study. For the follow-up study 193 women attended (78%). However, for statistical analyses of this follow-up study the women who had changed their HRT protocol (using/not-using HRT) between the baseline and 2-year follow-up were excluded (n = 32). Also women who had reported regular menstruation were excluded from the final analyses (n = 11). Finally the results from 161 were included in our analyses but complete microbiological results were available for only 135 subjects. The study profile is given in Figure 1.

Ethical approval of the study had been originally given by the City of Helsinki Health Department (ethical permit #53 1.4.1997). All subjects attending signed an informed consent according to the Declaration of Helsinki.

Use and duration of prescribed medications including HRT as well as illnesses diagnosed by physician were asked with open questions with a structured questionnaire. Use of intrauterine contraception was not considered as HRT. For the statistical analyses a woman was included into the HRT group if she had used HRT at least six months at the time of the questionnaire.

Both the periodontal examinations and microbiological samplings, at baseline and two years later, were made by one dentist (author L.T). Oral examinations were made according to the WHO guidelines [19,20]. Panoramic jaw radiographs were available of each subject at the clinical examination. Periodontal probing depths were determined at all mesial, distal, vestibular (buccal, labial), and oral (lingual, palatal) tooth surfaces for scoring the Community Periodontal Index of Treatment Needs (CPITN) [19]. The periodontal pocket depths were measured with a WHO probe (tip diameter 0.5 mm) to the nearest millimeter (mm) from the gingival margin to the bottom of the pocket. "Periodontitis" was diagnosed if at least one sextant had a CPITN - score 3 or higher, thus indicating at least ≥ 4 mm deep pockets in the subject. Individual pocket depths were not recorded separately. However, the number of ≥ 6 mm deep pockets was counted separately for each patient.

### Microbial sampling methods and analyses

Subgingival plaque from the four deepest periodontal sites was sampled in every patient. If no ≥ 4 mm pockets existed or there were less than four ≥ 4 mm pockets in the subject the rest of the plaque samples were taken from the first premolars. Before sampling the supragingival sites, plaque was gently removed with a cotton swab and the site of collection was isolated with cotton rolls and dried. Thereafter, the subgingival plaque samples were taken with a sterile curette and placed into a micro-centrifuge tube containing 0.5 ml distilled water. The pooled samples were then centrifuged and 5 μl aliquots of supernatant were added to the PCR reaction mixtures. The PCR method has been described in detail by Wahlfors et al. [21] and Meurman et al. [22]. Periodontal bacteria *A. actinomycetemcomitans, P. gingivalis, T. forsythia, T. denticola, P. intermedia *and *P. nigrescens *were analyzed.

### Statistical methods

Women using or not using HRT were analyzed separately and then the groups were compared in the 2-year follow-up scheme. Only those who were examined both at baseline and then followed-up for two years were included in the final analyses. Cross-tabulations and chi-square tests were used in the SPSS for Windows statistical program (SPSS Inc. Chicago, Ill., USA, version 13.0). Stepwise logistic regression analyses were made to study the explanatory factors for the positive periodontal microbial findings. The explanatory factors used in the backward stepwise logistic regression model were age, prevalence of cardiovascular disease, number of teeth, use of HRT, WHO diseased, missed, filled tooth index - score, existence of ≥ 4 mm deep and ≥ 6 mm deep periodontal pockets using the CPITN-score data, and self-assessed poor oral health. The effect of systemic diseases and medications on the results was analyzed separately for HRT and non-HRT groups using median values of the total number of concomitant illnesses and drugs used daily; both at baseline and two years later.

## Results

The reasons for drop-outs and exclusion from the study were mainly change in the original protocol by either starting HRT by women originally assigned to the non-HRT group or *vice versa*, or lack of interest. The demographic characteristics of the drop-outs and those excluded did not differ from the women who were eligible for the 2-year follow-up analyses. As expected, women not using HRT reported more often climacteric symptoms than HRT users: 35% at baseline *vs*. 13% at the 2-year examination, respectively (p < 0.001).

At baseline the percentage of women with periodontitis (defined as at least one sextant with CPITN score-3) was 79% in HRT group and 80% in non-HRT group. Two years later the respective figures were 71% and 76%. The mean numbers of ≥ 6 mm deep periodontal pockets were 0.9 ± 1.7 at baseline *vs*. 1.1 ± 2.1 two years later in the HRT group, and 1.0 ± 1.7 *vs*. 1.2 ± 1.9, respectively, in the non-HRT group. These differences were not statistically significant. More detailed data of the dental health parameters are described in Table 1.

**Table 1:** Descriptive data of the study subjects divided in groups according to the use of hormone replacement therapy (HRT).

	Baseline			Two-year follow-up		
	**HRT (106)**	**Non-HRT (55)**	**Significance** **between groups**	**HRT (106)**	**Non-HRT (55)**	**Significance between groups**

Mean age	55.4 ± 2.7	55.9 ± 2.4	ns.	57.4 ± 2.7	57.9 ± 2.4	ns.
Current smoker	23(22%)	11(20%)	ns.	19(18%)	8 (15%)	ns.
Current climateric symptoms	16 (15%)	24 (44%)	p < 0.001	14 (13%)	19 (35%)	p < 0.001
Satisfactory self-assesed dental health	65 (61%)	28 (51%)	ns.	63 (59%)	31 (56%)	ns.

**Diagnosed illness:**						

Cardiovascular disease	19 (18%)	10 (18%)	ns.	25 (24%)	11 (20%)	ns.
Psychiatric disease	1 (1%)	2 (4%)	ns.	1 (1%)	3 (6%)	ns.
Asthma	8 (8%)	3 (6%)	ns.	12 (11%)	3 (6%)	ns.
Rheumatic disease	8 (8%)	2 (4%)	ns.	6 (6%)	3 (6%)	ns.

**Regular medication:**						

Use of cardiovascular drugs#	29 (27%)	14 (26%)	ns.	39 (37%)	14 (26%)	ns.
Use of neurological drugs#	7 (7%)	10 (18%)	p < 0.05	5 (5%)	9 (16%)	p < 0.05
Use of respiratory drugs#	7 (7%)	4 (7%)	ns.	15 (14%)	4 (7%)	ns.
Use of analgesics and antipyretics	7 (7%)	1 (2%)	ns.	5 (5%)	5 (9%)	ns.
Allergy medication	4 (4%)	0 (0%)	ns.	0	0	-
Oestrogen therapy	49 (47%)			47 (47%)		
Progestin therapy	2 (2%)			1 (1%)		
Combination therapy	53 (51%)			53 (53%)		

Ns. = Statistical difference non-significant.

#Includes diuretics, antihypertensive agents, nitrates, digitalis and antiarrhythmic agents;

##Includes antidepressant drugs, tranquilizers, sedatives and antipsychotic agents;

### Mainly medication for asthma.

**Figure 2:**
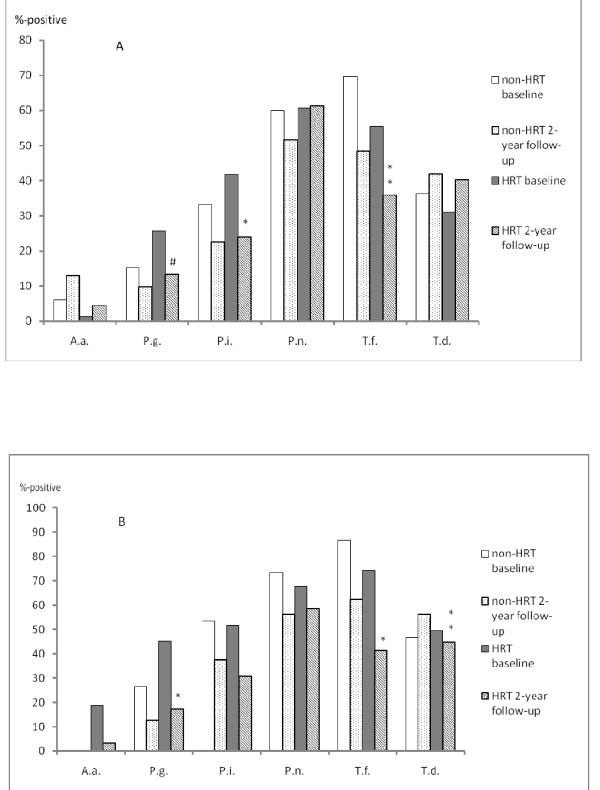
**Periodontal micro-organisms detected at baseline and two years later in the women with (HRT) or without (non-HRT) hormone replacement therapy**. **Graph A **shows the results of women with the Community Periodontal Index for Treatment Need (CPITN) score 3, indicating the existence of ≥ 4 mm deep periodontal pockets, and **graph B **of those with CPITN score 4, respectively, indicating ≥ 6 mm periodontal pockets. A.a. = Aggregatibacter actinomycetemcomitans, P.g. = Porphyromonas gingivalis, P.i. = Prevotella intermedia, P.n. = Prevotella nigrescens, T.f. = Tannerella forsythia, T.d. = Treponema denticola. The asterisks show statistically significant differences between baseline and follow-up samples (*p < 0.05, **p < 0.01; #p = 0.07).

Figure 2 gives the frequencies of the positive periodontal microbial results of the subjects with ≥ 4 mm and ≥ 6 mm deep periodontal pockets, respectively, as assessed using the CPITN scoring. In general, at baseline the periodontal pathogen *P. gingivalis *was detected in approximately 20% among all the women, while *A. actinomycetemcomitans *was detected in less than 5%. *T. forsythia *and *T. denticola *were detected in 30-50% of the women. There was no statistically significant difference in the frequency of positive microbial findings between the groups at baseline. However, two years later significantly less positive cases of *P. intermedia *(p < 0.05) and *T. forsythia *(P < 0.01) were detected in the samples from the ≥ 4 mm deep pockets of the HRT users while no such difference was observed in the non-HRT group. Of the HRT users the decrease in the number of positive samples of *P. gingivalis *almost reached statistical significance in the ≥ 4 mm deep pockets (P = 0.069). Correspondingly, the 2-year follow-up results of the samples from the ≥ 6 mm deep pockets of the women using HRT showed a significant decrease in the number of positive cases of *P. gingivalis *(p < 0.05) and *T. forsythia *(p < 0.05) while no difference was observed in the non-HRT group.

When the background variables for the prevalence of periodontal micro-organisms in general were analyzed in the stepwise logistic regression, the existence of deep periodontal pockets (data based on CPITN score 3 or higher) explained the majority of the cases. As regards the effect of reported systemic diseases on the microbiological results, *A. actinomycetemcomitans *was found to be more prevalent in women with cardiovascular disease (Odds ratio [OR 5.48], 95% confidence interval [CI] 1.04 - 29.77; p < 0.05). No other bacterial species investigated associated statistically with any systemic disease reported. However, in general the number of these cases was only a few individuals, so the finding on *A. actinomycetemcomitans *probably has no clinical relevance.

Similarly to the results of the prevalence of reported systemic diseases *vs*. positive bacterial samples analyses of the associations between the use of drugs and the microbiological results showed that in addition to hormones in the HRT group other medication was rare in the subjects. At baseline the mean number of daily used drugs was 0.7 in all the women while two years later the respective figures were 0.5 drugs in the HRT group and 0.7 drugs in the non-HRT group. At the 2-year examination, women of the non-HRT group reported more often use of neurological drugs than those of the HRT group (16% *vs*. 5% p < 0.01). In the non-HRT group *T. forsythia *was detected more often among women not using any systemic drugs (73.7% *vs*. 26.3%, p < 0.05). Again, however, the number of the women in these subgroups analyzed was small and, subsequently, the findings may not have clinical relevance.

In both groups the main explanatory factor in the model for the occurrence of *P. gingivalis, P. intermedia, T. forsythia *and *T. denticola *was the existence of deep periodontal pockets. The results of the stepwise logistic regression analyses are given in detail in Table 2.

**Table 2:** Odds ratios (OR) with 95% confidence intervals (95% CI) of the statistically significant explanatory factors after backward elimination for the prevalence of periodontal micro-organisms analyzed by stepwise logistic regression.

Bacterium#	Explanatory factor§	OR	95% CI	Significance
Baseline				
P.g.	Periodontal pockets	3.73	1.74-8.00	p = 0.001
P.i.	Periodontal pockets	2.77	1.57-4.91	p < 0.001
P.n.	Poor oral health	0.40	0.19-0.82	p = 0.012
T.f.	Periodontal pockets	4.44	2.44-8.07	p < 0.001
T.d.	Periodontal pockets	2.26	1.28-3.97	p = 0.005

**2 year-Follow-up**				

A.a.	HRT§§	0.21	0.48-0.93	p = 0.040
	Cardiovascular			
	medication§§§	5.48	1.04-28.77	p = 0.044
P.g.	Periodontal pockets	3.04	1.11-8.34	p = 0.031
P.i.	Periodontal pockets	1.98	1.09-3.59	p = 0.025
P.n.	Number of teeth	1.08	1.00-1.16	p = 0.045
T.d.	Periodontal pockets	2.95	1.06-8.19	p = 0.038

#A.a. = *Aggregatibacter actinomycetemcomitans*; P.g. = *Porphyromonas gingivalis*;

P.i. = *Prevotella intermedia*; P.n. = *Prevotella nigrescens*; T.f. = *Tannerella forsythia*; T.d. = *Treponema denticola*.

§Periodontal pockets: >4 mm deep pockets; Poor oral health = self-assessed poor oral health;

§§HRT = hormone replacement therapy

§§§Includes diuretics, antihypertensive agents, nitrates, digitalis and antiarrhythmic agents

## Discussion

We observed that the number of *T. forsythia- *positive samples decreased significantly during the 2-year follow-up in both study groups while the decrease in the number of *P. gingivalis - *positive samples was significant only in the HRT users, in particular in women with ≥ 6 mm deep periodontal pockets. A significant decrease was also observed in the *P. intermedia*-positive samples from women of the HRT group with ≥ 4 mm deep periodontal pockets. No such differences were observed in the non-HRT group. These findings partly support our study hypothesis, namely that HRT use may affect periodontal infections, since a decrease was observed specifically in the number of positive samples for the pathogens *T. forsythia *and *P. gingivalis*.

Because our follow-up time was only two years no significant difference was expected in the clinical periodontal status between the women using or not using HRT. As earlier reported this was indeed the case in the present study cohort [17]. The strongest explanatory factor for harboring the bacteria investigated was the existence of deep periodontal pockets in both study groups. This result came as no surprise since periodontal pockets and furcation lesions are obviously the characteristic sites harboring periodontal pathogens. Similarly, self-assessed poor oral health status associated with harboring the bacteria investigated but in the present study only the less harmful *P. nigrescens - *species linked statistically with this parameter. *P. nigrescens *has been associated with gingivitis [23], but it is also frequently isolated from periodontally healthy sites similar to the *P. intermedia *species [24]. Since we used pooled plaque samples there are no data from individual periodontal pockets. Our aim was rather to assess the overall effect of HRT on periodontal microbiota than monitoring single pockets in this respect. In the retrospect, it might have been interesting to monitor the periodontal bacteria at individual pockets. Taken together, however, more long-term investigations are needed for confirming the present results and for the final evaluation of the effect of HRT on periodontal micro-organisms.

It is worth a notion that oral micro-organisms have been shown to metabolize *in vitro *steroid hormones [25,26]. The metabolism of progesterone, for example, has been shown to increase in inflamed gingival tissue [27]. Of the bacterial species analyzed in our study, *T. denticola *has been shown to utilize host-derived steroids as growth factors which phenomenon may link to its virulence [28]. Hence, in light of these metabolic findings one might have expected increased *T. denticola *counts during the 2-year observation which is contrary to our observation. The present results illustrate the complexity of the interactions between steroid hormones, periodontal tissue and micro-organisms. There is a need for more in-depth studies also in this area.

Earlier investigations have shown that in menopause the risk for tooth loss is smaller in women using HRT [29]. The circulating levels of estrogen influence alveolar bone density so that more periodontal lesion sites showed loss in alveolar bone density if serum estradiol level was suboptimal [30]. In our study the women using HRT had used it at least for 6 months already in the beginning of the investigation so they were in the steady state regarding hormone balance. However, Pilgram et al. [31] investigated 135 women in a randomized controlled trial with estrogen replacement for three years and did not find statistically significant changes in clinical attachment of teeth and bone mineral density of lumbar spine. A study from Spain, on the other hand, showed improvement in periodontal probing depths and tooth mobility in 190 women randomized to receive HRT for one year [32]. However, there are no systematic studies on the effect of different hormone combinations on periodontal micro-organisms and oral health parameters. It can be assumed that different hormone combinations may also affect periodontal tissues and microbiota in a highly specific way.

To our knowledge, the present study was the first follow-up study where periodontal micro-organisms in general have been investigated in clearly defined age groups of peri-menopausal and postmenopausal women. Norderyd and coworkers reported earlier that postmenopausal women taking estrogen supplementation had more frequent gingival bleeding than control group, but they did not find significant differences in the levels of periodontal pathogens examined except for *Capnocytophaga *-species which are usually implicated in the initiation of puberty gingivitis [12,14,33]. The strength of the present study was the fact that the subjects were originally recruited from a large group of women invited for free mammography examination in age groups of 50-58 years [17,18]. Since the examination was free-of-charge it can be anticipated that selection bias was minimal. However, there is always the possibility that women less interested in their health did not come, but the same holds true for both those using and not using HRT. The weakness of the study was the fairly short 2-year follow-up period. Hence big changes in oral health parameters were not to be expected. For practical reasons the protocol had to be time-restricted and a 2-year longitudinal study was possible as also conducted earlier by Grodstein et al. [29]. Another weakness was the drop-out of subjects which was mainly due to the reason that women originally not using HRT had started the therapy and those in the HRT group had ceased using hormones during the 2-years of follow-up. They were then excluded from the statistical analyses.

Finally, the observation that certain periodontal pathogens decreased in the HRT group during the 2-year follow-up might also be due to increased health consciousness of the participants. The women might have improved their oral hygiene habits after oral hygiene information given at the baseline clinical examination. Thus an unavoidable bias caused by trial effect might modify the results in this respect.

## Conclusion

To conclude, we observed a decreased number of *P. gingivalis- *and *T. forsythia- *positive samples in women of the HRT group during the 2-year follow-up. The findings partly support our study hypothesis, namely that HRT use may affect periodontal infections. Clinical relevance of the results needs to be assessed in future studies with longer observation time, however.

## Competing interests

The authors declare that they have no competing interests.

## Authors' contributions

LT participated in study design and carried out the study and wrote the manuscript. KK was in charge of the microbiological analyses, JF performed and interpreted the statistical analyses, AT and JHM supervised the study, interpreted the data and contributed to drafting the manuscript. All authors read and approved the final manuscript.

## Pre-publication history

The pre-publication history for this paper can be accessed here:

http://www.biomedcentral.com/1472-6831/10/10/prepub

## References

[B1] Grodstein F Stampfer R The epidemiology of coronary heart disease and estrogen replacement in postmenopausal women Prog Cardiovasc Dis 1995 38 199 221 10.1016/S0033-0620(95)80012-3 7494882

[B2] Seeman E Tsalamandris C Bass S Pearce G Present and future osteoporosis therapy Bone 1995 17 23 29 10.1016/8756-3282(95)00203-P 8579894

[B3] Herrington DM Reboussin DM Brosnihan KB Sharp PC Shumaker SA Snyder TE Furberg CD Kowalchuk GJ Stuckey TD Rogers WJ Givens DH Waters D Effects of estrogen replacement on the progression of coronary-artery atherosclerosis N Engl J Med 2000 343 522 529 10.1056/NEJM200008243430801 10954759

[B4] Hulley S Grady D Bush T Furberg C Herrington D Riggs B Vittinghoff E Randomized trial of estrogen plus progestin for secondary prevention of coronary heart disease in postmenopausal women. Heart and estrogen/progestin replacement study (HERS) research group J Am Med Assoc 1998 280 605 613 10.1001/jama.280.7.605 9718051

[B5] Hsia J Langer RD Manson JE Kuller L Johnson KC Hendrix SL Pettinger M Heckbert SR Greep N Crawford S Eaton CB Kostis JB Caralis P Prentice R for the Women's Health Intiative Investigators Conjugated equine estrogens and coronary heart disease: The Women's Health Intiative Arch Intern Med 2006 166 357 365 10.1001/archinte.166.3.357 16476878

[B6] Manson JE Hsia J Johnson KC Rossouw JE Assaf AR Lasser NL Trevisan M Black HR Heckbert SR Detrano R Stirckland Wong ND Crouse JR Stein E Cushman M for the Women's Health Intiative Investigators Estrogen plus progestin and the risk of coronary heart disease N Engl J Med 2003 349 2075 2076 10.1056/NEJMoa030808 12904517

[B7] Caufriez A Hormonal replacement therapy (HRT) in postmenopause: a reappraisal Ann Endocrinol 2007 68 241 250 10.1016/j.ando.2007.06.015 17651686

[B8] Harman SM Estrogen replacement in menopausal women: recent and current prospective studies, the WHI and the KEEPS Gend Med 2006 3 254 269 10.1016/S1550-8579(06)80214-7 17582367

[B9] Friedlander AH The physiology, medical management and oral implications of menopause J Am Dent Assoc 2002 133 73 81 11811747 10.14219/jada.archive.2002.0025

[B10] Meurman JH Sanz M Janket SJ Oral health, atherosclerosis and cardiovascular disease Crit Rev Oral Biol Med 2004 15 403 413 10.1177/154411130401500606 15574681

[B11] Pihlstrom BL Michalowicz BS Johnson NW Periodontal diseases Lancet 2005 366 1809 1820 10.1016/S0140-6736(05)67728-8 16298220

[B12] Norderyd OM Grossi SG Machtei EE Zambon JJ Hausmann E Dunford RG Genco RJ Periodontal status of women taking postmenopausal estrogen supplementation J Periodontol 1993 64 957 62 8277404 10.1902/jop.1993.64.10.957

[B13] Sooriyamoorthy M Gower DB Hormonal influences on gingival tissue: relationship to periodontal disease. Review Article J Clin Periodontol 1989 16 201 208 10.1111/j.1600-051X.1989.tb01642.x 2654195

[B14] Mascarenhas P Gapski R Al-Shammari K Wang H-L Influence of sex hormones on the periodontium. Review Paper J Clin Periodontol 2003 30 671 681 10.1034/j.1600-051X.2003.00055.x 12887335

[B15] Brennan RM Genco RJ Wilding GE Hovey KM Trevisan M Wactawski-Wende J Bacterial species in subgingival plaque and oral bone loss in postmenopausal women J Periodontol 2007 78 1051 1061 10.1902/jop.2007.060436 17539719

[B16] Carrillo-de-Albornoz A Figuero A Herrera D Bascones-Martinez A Gingival changes during pregnancy: II. Influence of hormonal variations on the subgingival biofilm J Clin Periodontol 2010 37 230 240 10.1111/j.1600-051X.2009.01514.x 20088983

[B17] Tarkkila L Furuholm J Tiitinen A Meurman JH Oral health in perimenopausal and early post menopausal women from baseline to 2 years of follow-up with reference to hormone replacement therapy Clin Oral Invest 2008 12 271 277 10.1007/s00784-008-0190-z 18299902

[B18] Tarkkila L Linna M Tiitinen A Lindqvist C Meurman JH Oral symptoms at menopause-the role of hormone replacement therapy Oral Surg Oral Med Oral Path Oral Radiol Endod 2001 92 276 280 10.1067/moe.2001.117452 11552144

[B19] Ainamo J Barmes D Beagrie G Cutress T Martin J Sardo-Infirri J Development of the World Health Organization (WHO) Community periodontal index of treatment need (CPITN) Int Dent J 1982 32 281 291 6958657

[B20] World Health Organization Oral health surveys 1997 5 WHO, Geneva

[B21] Wahlfors J Meurman JH Vaisanen P Alakuijala P Korhonen A Torkko H Janne J Simultaneous detection of Actinobacillus actinomycetemcomitans and Porphyromonas gingivalis by a rapid PCRmethod J Dent Res 1995 74 1796 1801 10.1177/00220345950740111301 8530743

[B22] Meurman JH Wahlfors J Korhonen A Alakuijala P Vaisanen P Torkko H Janne J Identification of Bacteroides forsythus in subgingival dental plaque with the aid of a rapid PCR method J Dent Res 1997 76 1376 1380 10.1177/00220345970760070701 9207770

[B23] Lie MA Weijden GA van der Timmerman MF Loos BG van Steenbergen TJ Velden U van der Occurrence of Prevotella intermedia and Prevotella nigrescens in relation to gingivitis and gingival health J Clin Periodontol 2001 28 189 193 10.1034/j.1600-051x.2001.028002189.x 11168745

[B24] Paster BJ Boches SK Galvin JL Ericson RE La CN Levanos VA Sahasrabudhe A Dewhirst FE Bacterial diversity in human subgingival plaque J Bacteriol 2001 183 3770 3783 10.1128/JB.183.12.3770-3783.2001 11371542 PMC95255

[B25] Ojanotko-Harri A Laine M Tenovuo J Metabolism of 17 beta-estradiol by oral Streptococcus mutans, Streptococcus sanguis, Bacillus cereus and Candida albicans Oral Microbiol Immunol 1991 6 126 128 10.1111/j.1399-302X.1991.tb00465.x 1945489

[B26] Soory M Targets for steroid hormone mediated actions of periodontal pathogens, cytokines and therapeutic agents: some implications on tissue turnover in the periodontium Curr Drug Targets 2000 1 309 325 10.2174/1389450003349119 11467074

[B27] Ojanotko-Harri A Metabolism of progesterone by healthy and inflamed human gingiva in vitro J Steroid Biochem Mol Biol 1985 23 1031 1035 10.1016/0022-4731(85)90063-9 4094411

[B28] Clark DT Soory M The metabolism of cholesterol and certain hormonal steroids by Treponema denticola Steroids 2006 71 352 63 10.1016/j.steroids.2005.11.006 16436288

[B29] Grodstein F Colditz GA Stampfer MJ Post-menopausal hormone use and tooth loss: a prospective study J Am Dent Assoc 1996 127 370 377 8819784 10.14219/jada.archive.1996.0208

[B30] Payne JB Zachs NR Reinhardt RA Nummikoski PV Patil K The association between estrogen status and alveolar bone density changes in postmenopausal women with a history of periodontitis J Periodontol 1997 68 24 31 9029448 10.1902/jop.1997.68.1.24

[B31] Pilgram TK Hildebolt CF Dotson M Cohen SC Hauser JF Kardaris E Civitelli R Relationships between clinical attachment level and spine and hip bone mineral density: data from healthy postmenopausalwomen J Periodontol 2002 73 298 301 10.1902/jop.2002.73.3.298 11922259

[B32] Lopez-Marcos JF Garcia-Valle S Garcia-Iglesias AA Periodontal aspects in menopausal women undergoing hormone replacement therapy Med Oral Patol Oral Cir Bucal 2005 10 1321 1341 15735546

[B33] Mombelli A Lang NP Burgin WB Gusberti FA Microbial changes associated with the development of puberty gingivitis J Periodontal Res 1990 25 331 338 10.1111/j.1600-0765.1990.tb00924.x 2148945

